# Data Perturbation Independent Diagnosis and Validation of Breast Cancer Subtypes Using Clustering and Patterns

**Published:** 2007-02-19

**Authors:** G. Alexe, G.S. Dalgin, R. Ramaswamy, C. DeLisi, G. Bhanot

**Affiliations:** 1 Computational Biology Center, IBM Thomas J. Watson Research Center, Yorktown Heights, NY 10598, U.S.A; 2 The Simons Center for Systems Biology, Institute for Advanced Study, Princeton NJ 08540, U.S.A; 3 Molecular Biology, Cell Biology and Biochemistry Program, Boston University, 2 Cummington Street, Boston, MA 02215, U.S.A; 4 School of Information Technology, Jawaharlal Nehru University, New Delhi 110 067, India; 5 Biomedical Engineering, Boston University, 44 Cummington Street, Boston, MA 02215, U.S.A; 6 Department of Biomedical Engineering and BioMaPS Institute, Rutgers University, Piscataway, NJ 08854

**Keywords:** Breast cancer, Clusters, Patterns, Multi-gene Biomarkers, Diagnosis

## Abstract

Molecular stratification of disease based on expression levels of sets of genes can help guide therapeutic decisions if such classifications can be shown to be stable against variations in sample source and data perturbation. Classifications inferred from one set of samples in one lab should be able to consistently stratify a different set of samples in another lab. We present a method for assessing such stability and apply it to the breast cancer (BCA) datasets of Sorlie et al. 2003 and Ma et al. 2003. We find that within the now commonly accepted BCA categories identified by Sorlie et al. Luminal A and Basal are robust, but Luminal B and ERBB2+ are not. In particular, 36% of the samples identified as Luminal B and 55% identified as ERBB2+ cannot be assigned an accurate category because the classification is sensitive to data perturbation. We identify a “core cluster” of samples for each category, and from these we determine “patterns” of gene expression that distinguish the core clusters from each other. We find that the best markers for Luminal A and Basal are (ESR1, LIV1, GATA-3) and (CCNE1, LAD1, KRT5), respectively. Pathways enriched in the patterns regulate apoptosis, tissue remodeling and the immune response. We use a different dataset (Ma et al. 2003) to test the accuracy with which samples can be allocated to the four disease subtypes. We find, as expected, that the classification of samples identified as Luminal A and Basal is robust but classification into the other two subtypes is not.

## Introduction

Breast cancer (BCA) is a common and heterogeneous disease affecting women of all ages. Its occurrence is correlated with levels of estrogen (ER), progesterone (PR) and Her2neu (ERBB2) (Gruvberger et al. 2001; Lacroix and Leclercq 2005). Clinically, BCA is classified into two major subtypes: ER+ and ER−. These groups are sometimes stratified further by ERBB2 and/or PR levels. Across all treatments, ER+ and/or PR+ patients have a better prognosis than ER− and/or PR− tumors (Anim et al. 2005) and are also more likely to respond to hormone therapy (e.g. tamoxifen). Over-expression of ERBB2, seen in 25–30% of cases, is often a marker of aggressive disease, poor prognosis and mixed treatment results (Diermeier et al. 2005).

In spite of sustained research and medical and pharmaceutical effort, the incidence and death rate of BCA remains high. In 2005, more than 1.2 million new cases were diagnosed world wide and more than 20% of these will die from the disease (http://imaginis.com/breasthealth/). A major cause of treatment failure is that tumors with similar histopathology have divergent clinical courses and prognoses. The goal of the present study is the same as that of many others (Bieche et al. 1995; West et al. 2001; van’t Veer et al. 2002; Honig et al. 2004; Ahnstrom et al. 2005; Sharma et al. 2005; Osipo et al. 2005), that molecular profiling of BCA will clarify molecular correlates of disease, and this in turn will improve choice of therapy, and provide leads to new and more effective therapeutics.

In a series of papers on analysis of cDNA data of BCA tissue samples (Sorlie et al. 2001; Perou et al. 2000, 2001) the samples were uniquely assigned to one of four distinct categories: Luminal A, Luminal B, ERBB2+ (or Her2+) and Basal-like. These subtypes were later validated by Sotiriou et al. 2003, Loi et al. 2005 and Kristensen et al. 2005. The first two categories were mostly ER+ and the latter two mostly ER− negative. In the original analysis of Perou et al. 2000, Basal tumors were characterized by high levels of keratins 5 and 17, laminin, and fatty acid binding protein 7 genes (see also Charafe-Jauffret et al. 2005), whereas ERBB2+ was characterized by high levels of several genes in the ERBB2 amplicon at 17q12.21 including ERBB2 and GRB7. Other studies identified different markers (Abd El-Rehim et al. 2005; Bertucci et al. 2005; Farmer et al. 2005; Hu et al. 2006; Sorlie et al. 2006) and a consensus set of markers for all BCA patients is not currently available.

Luminal and Basal-like tumors arise in distinct breast tissue cell types (Perou et al. 2000) and have very different disease course (Sorlie et al. 2001, 2003) and response to therapeutics (Troester et al. 2004; Bertucci et al. 2005). The Luminal A subtype has the best overall prognosis followed by Luminal B while the other two subtypes are more aggressive and difficult to treat. The nomenclature of these subtypes has found its way into the language and culture of clinical practice and affects treatment options offered to patients. This makes it important to validate the stability of the original classification of Sorlie et al. This is the main goal of the present paper.

The original analysis used simple hierarchical clustering (Eisen et al. 1998) which is known to be sensitive to data perturbation (Monti et al. 2003; van der Kloot et al. 2005). We re-analyzed the data using a robust averaging procedure to access the stability of imposing five clusters (4 disease subtypes + Normal) on the data. The goal was to identify a “core” set of samples in each subtype which were stable under data perturbations, and to use these cores to determine “patterns” of gene expression for each core. We found stable core clusters for samples in the Luminal A, Basal and Normal clusters of the original analysis. However, the “Luminal B” and “ERBB2+” clusters of Sorlie et al. were unstable, with only a subset of the samples from the previous assignment remaining in stable core clusters under data perturbation. Instead, the originally assigned samples scattered over two or more clusters. This suggests that the Luminal B and ERBB2+ clusters (and their markers) as identified in Sorlie et al. 2003, are unstable to data perturbation and need further analysis.

For the Luminal A and Basal categories, we find a robust set of gene markers and patterns. If we combine the Sorlie et al. dataset with a new dataset from Ma et al. and cluster the combined data using these robust gene markers and patterns, then in the new data, we can assign a robust subtype label for Luminal A and Basal but not for the other two disease phenotypes.

## Materials and Methods

### Datasets

#### Data 1

The cDNA dataset of (Sorlie et al. 2003) was obtained from http://genome-www.stanford.edu/breast_cancer/robustness/data/SupplText.html. The data had expression levels of *N* = 552 genes for *M* = 122 samples of which 112 were from BCA patients and 10 controls. The 552 genes were selected by Sorlie et al. to have small variation in tissue samples from the same patient and a high variation in tissue samples from different patients.

#### Data 2

The Ma et al. dataset was downloaded from www.geneexpression_ma.org. It consisted of expression levels of 1940 genes for 93 samples micro-dissected from 36 BCA patients and 3 normals. The samples were from three stages of disease: atypical ductal hyperplasia or ADH, ductal carcinoma *in situ* or DCIS and invasive ductal carcinoma or IDC respectively. The genes made available in the data were chosen by linear discriminant analysis as markers for breast cancer progression. ER, PR and HER2neu levels measured through immunohistochemistry were available.

### Preprocessing and Imputation for Data 1

The matrix of samples (columns) and genes (rows) was normalized to mean 0 and variance 1 first across columns and then across rows, ignoring missing entries. The matrix had 5,027 missing entries. We first eliminated genes and samples with more than 20% missing entries. This reduced the data to *N* = 530 genes and *M* = 118 samples. We imputed the missing entries using a simple generalization of the *k*NN method of Troyanskaya et al. 2001 as follows:

We identified the *k* nearest neighbor entries for missing entry *x_ij_* using the Euclidean metric,

$$d(i,i^{\prime })={\left(\sum_{j}{\left(x_{ij}-x_{i^{\prime }j}\right)}^{2}\right)}^{1/2}$$

with the requirement that the genes chosen as nearest neighbors have at least *t*% filled entries. Twenty imputations were done at each *x_ij_* using the range 10 ≤ *k* ≤ 14 for *k* and varying *t* from 50% to 80% in increments of 10. Let {*x*_1_, *x*_2_, …, *x_k_*} be the *k*-nearest neighbor entries in increasing order of distance and *R* be a uniform random number in (0,1). Then the imputed value

*y* is given by *y* = *x_j_*, which satisfies

$$\begin{array}{c} \sum_{i=1}^{j-1}\frac{x_{i}}{X}<R\leq \sum_{i=1}^{j}\frac{x_{i}}{X},\\ \text{where }X=\sum_{i=1}^{k}x_{i}. \end{array}$$

Twenty datasets were generated in this way, one for each (*k*,*t*) value. The clustering was averaged over these twenty datasets in order to create a set of clusters insensitive to parameter choice in data imputation. This averaging is an improvement over the *k*NN method because it is stable to both variation in *k* and variation in how the neighbors are chosen (as measured by *t*). Multiple clones in the data were eliminated by averaging after discarding outliers outside a 95% confidence interval. This process left 523 genes with no missing entries or clones. The final data is given in Supplementary Table 1.

## Results

### Identifying “Core” Clusters

We use the letters A, B, C, D, E to denote the five phenotypes: Luminal A, Luminal B, ERBB2+, Basal, and Normal respectively. The clusters were identified using the consensus hierarchical clustering technique of Monti et al. 2003 implemented in GenePattern (http://www.broad.mit.edu/cancer/software/genepattern/). This method assesses the stability of hierarchical clustering across multiple perturbations of the data. We generated 100 copies of the dataset by randomly selecting 80% of the samples. Each copy was hierarchically clustered using a Euclidean distance metric and the top 5 clusters were selected. For each distinct sample pair (*i, j*) in the data, we computed the frequency *F_ij_* with which the pair clustered together over the 100 copies of the datasets. The matrix of *F_ij_* values is called the “agreement matrix.” Repeating this for all 20 data imputations and averaging gave the final “consensus agreement matrix” which is shown in Supplementary Table 2.

The five core clusters were identified as bicliques (Alexe et al. 2004) using the agreement matrix entries as a measure of similarity. We used the criterion that two samples have the same phenotype and belong to the same core cluster if they have a consensus agreement matrix score greater than *P*. For the Luminal A and Basal subtypes, the value *P* = 90% was sufficient to get an exact match between the core cluster identified by us and the assignment in Perou et al. 2000 and Sorlie et al. 2003. However, for samples assigned to Luminal B and ERBB2+ by the earlier study, these thresholds needed to be lowered to 50% and 25% respectively to get agreement with the previous assignments, suggesting that these categories are considerably less stable to data perturbation. The five core clusters contained 60 out of the 118 samples.

From the *F_ij_* values, we define the average agreement score between a sample *i* and other samples *j* in a given cluster *C* as

$$F_{i,C}=\frac{\sum_{j=1}^{n}F_{ij}}{n},$$

where *j* = 1, ... , *n*, and *n* is the number of samples in the cluster *C. F_i,C_* was calculated for each of the five clusters. The results are shown in Figures 1a–e. For each phenotype, we used a cutoff criterion on *F_i,C_* to assign it to the corresponding core cluster and these samples are shown in color. Many samples earlier identified as Luminal B also have a high score in our Basal core cluster (Figure 1b and 1d). This suggests that the Luminal B identification is problematic. Figure 1e also shows that some samples identified earlier as Luminal A are placed in our “Normal” core cluster, suggesting that these patients may have minimal disease. Overall, our analysis shows that Luminal A, Basal and Normal phenotypes are robustly classifiable into homogeneous clusters but Luminal B and ERBB2+ do not cluster well. We find that 36% of the samples previously placed in the Luminal B category and 55% of samples previously classified as ERBB2+ are in fact ambiguous; i.e., their assignments are highly sensitive to data perturbation and they should be reanalyzed or classified as ambiguous. The scores of some unclassified samples in Sorlie et al. 2003 are shown in Figure 1f. For the samples where these scores are higher than the cutoff in one core cluster but not in any other, the corresponding sample can be assigned a category label by our clustering.

Table 1 compares the original assignments of Sorlie et al. with our core clusters of Figure 1 and shows the sample id’s from the original study.

The agreement fraction between the original assignment and our assignments is highest for the Normal, Luminal A and Basal categories and lower in the other two phenotypes.

For each sample *i* in a core cluster, we also calculated the silhouette score (Rousseeuw,1987) defined by

$$s(i)=s(i)=\frac{b(i)-a(i)}{\text{max}(a(i),b(i))},$$

where *a*(*i*) is the average dissimilarity between *i* and all other samples in the cluster, and *b*(*i*) is the minimum average dissimilarity of *i* to all samples in other clusters. If *s*(*i*) values in a cluster are close to unity, the cluster is well defined. An *s*(*i*) value near zero indicates that the sample is between two clusters. Negative values of *s*(*i*) mean that the sample is in the wrong cluster. The “silhouette width” of a cluster is the average of the *s*(*i*) scores of all samples in that cluster. The silhouette widths for our core clusters as well as for the Sorlie et al. clusters are given in Table 1. The low values of the average silhouette scores are worrisome. They suggest either that the stratification into these phenotypes is problematic or that a better choices of genes is necessary to separate the phenotypes more reliably.

### Identifying Robust Gene Markers

Microarray datasets suffer from an overabundance of genes, most of which do not contribute to the signal. Identifying differentially expressed genes for a given set of phenotypes is a difficult problem for which many methods have been proposed. These can be divided into two major groups (Guyon and Ellisseeff, 2003, Inza et al. 2004, Lai et al. 2006, Jeffery et al. 2006) for supervised learning:

#### (i) Filtering or Variable Ranking methods

These select features based on quality scores. They include the fold change test (e.g. Mutch et al. 2002; Breitling and Herzyk, 2005), the t-test (Gossett, 1908, Tusher et al. 2001), the Wilcoxon-Mann-Whitney test (Bradley, 1968; Lehman, 1975), the Signal-to-Noise Ratio (SNR) test (Golub et al. 1999), the J5 test (Patel and Lyons-Weiler, 2004), the D1 test (Patel and Lyons-Weiler, 2004) etc. Another set of methods measure the “separability” of data into different phenotype classes. These include simple separability (Patel and Lyons-Weiler, 2004), weighted separability (Patel and Lyons-Weiler, 2004), envelope eccentricity (Alexe et al. 2006), separation measure (Alexe et al. 2006b) etc. A third class uses information-theoretic methods such as the entropy criterion (e.g. Furlanello et al. 2003; Liu et al. 2005), mutual information (e.g. Tourassi et al. 2001), information gain (Liu, 2004) etc. Finally, there are the statistical impurity measures (Su et al. 2003) which include the two-ing rule, the Gini index, max-minority, sum-minority, sum-of-variances etc.

#### (ii) Feature Subset Selection Methods

One such method selects those features which are useful for classification for a given machine learning algorithm (e.g. SVM (Vapnik, 1998), ANN (Bishop, 1995), kNN (Ripley, 1996) etc). More sophisticated approaches are embedded methods which include the selection of features as part of the training process for the classifier. These methods are computationally intensive and require efficient search strategies or a preliminary filtering of the non-reliable genes to reduce the dimensionality of the problem.

The existence of such a variety of feature selection methods poses a challenge in microarray data analysis. There have been recent attempts to combine various approaches into a meta selection procedure based on “majority-voting” using ranking by predictive content across many data perturbations and machine learning methods (e.g. Bhanot et al. 2005; Alexe et al. 2005a). Several studies (Guyon and Ellisseeff, 2001; Alexe et al. 2005b) have shown that variables which are only weakly correlated with phenotype are very useful when used in combinations. This principle has lead to the development and study of combinatorial markers or patterns (Crama et al. 1988; Bhanot et al. 2005; Alexe et al. 2006b).

In the present study, we have chosen to use a single feature selection method (namely the SNR test, Golub et al. 1999) which has been shown (Alexe et al. 2006b) to have good performance on genomic and proteomic data. However, we cannot guarantee that it is the best method, particularly because of the need to impute the missing data in the dataset of Sorlie et al. As an added check on the feature selection, we also use the combinatorial “pattern” method and averaging over data perturbations to reduce the errors from potentially “less than optimum” choice of features.

We identified a large pool of uni-gene markers for each core that distinguish it from the others using the signal-to-noise statistic. For gene *i*, if μ_1_(*i*) and μ_2_(*i*) be the average gene expression levels for the core and its complement and σ_1_(*i*) and σ_2_(*i*) the corresponding standard deviations, the signal-to-noise ratio (SNR) is defined as SNR = (μ_0_ – μ_1_)/(σ_0_ + σ_1_). The t-test statistic is the same as the SNR except that the denominator is (σ_0_^2^ + σ_1_^2^)^1/2^. Since (σ_0_ + σ_1_) > (σ_0_^2^ + σ_1_^2^)^1/2^ SNR is a more conservative criterion than the t-test.

The SNR statistic is preferred over the t-test in situations when the sample size in a class is small (less than 30) because it does not assume a Gaussian distribution for the underlying variables; an assumption which is implicit in the t-test. When combined with a permutation test for measuring p-values, the SNR statistic is a powerful and widely used technique for feature selection and class discrimination (e.g. Golub et al. 1999; Ramaswamy et al. 2001; Shipp et al. 2002; Sun et al. 2004; Goh and Kasabov 2005; Monti et al. 2005) and is implemented in several software packages (e.g. GenePattern and Gene Set Enrichment Analysis (GSEA), http://www.broad.mit.edu/tools/software.html).

The signal-to-noise (SNR) was computed for each gene for each of the 20 imputed datasets and for each of the 60 leave-one-out sample perturbation experiments for the core samples. The selected genes were those whose *p*-value for the SNR was below 0.01 and the significance of the SNR for false discovery rate (FDR) (Benjamini and Hochberg, 1995) was above 0.95 in each experiment.

This procedure identified 391 robust uni-gene markers (given in Supplementary Table 3) for the five core clusters. They consisted of overlapping sets of genes, 238 for Luminal A, 234 for Basal, 66 genes for Luminal B, 35 genes for ERBB2+ and 118 genes for Normals. These included many genes identified in previous studies (Perou et al. 2000; Sorlie et al. 2003; Loi et al. 2005). For example, the Luminal A set included the known estrogen pathway genes (ESR1, LIV1, GATA-3) and the Basal set the known genes CCNE1, LAD1, and KRT5.

**Figure 2:**
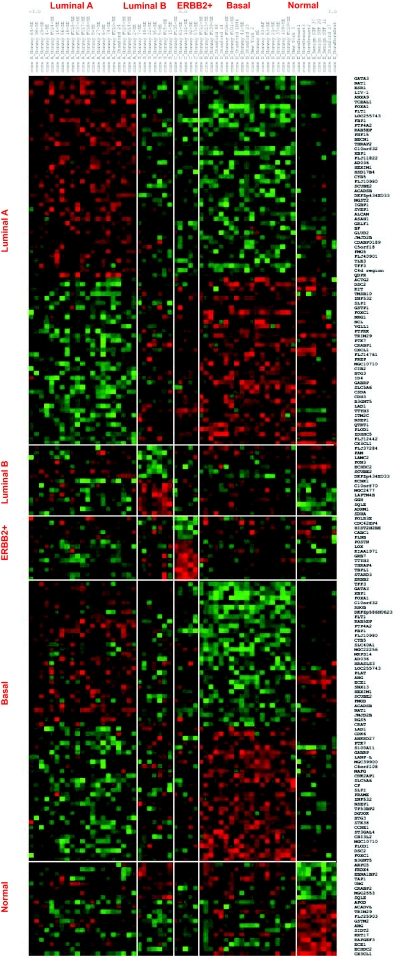
Heatmap of 148 uni-genes for the samples in core categories.

We further reduced this pool to 148 genes using the more stringent criteria which used the significance of the SNR for several metrics: the false discovery rate, the Q value (Storey and Tibshirani, 2003), FWER (Dudoit et al. 2002), Bonferroni correction (Bonferroni, 1935). More details about the multiple testing metrics we used are given in Supplementary Information I. These 148 genes included 79 genes for Luminal A and 60 for Basal with an overlap of 31 genes. The other phenotypes (Luminal B, ERBB2+ and Normal) had far fewer gene markers (15 for Luminal B, 14 for ERBB2+ and 20 for Normal core clusters). These genes are listed in Tables 2 a–d and those also identified in Sorlie et al. (2003) are marked with a*. A heat map of the core clusters using these 148 genes is shown in Figure 2.

### Patterns (Multi-gene Markers) for the Core Clusters

The complexity of BCA makes it unlikely that single genes can predict phenotype. Instead, one expects combinations of genes to be better at identifying phenotype. Consequently, we used “ patterns” (as defined in Crama et al. 1988; Alexe and Hammer, 2005; Bhanot et al. 2005) to distinguish the core clusters. A pattern is a set of linear constraints on the expression levels of a group of genes satisfied by many samples in a particular cluster and by few samples in other clusters. For example, the pattern ***PA*** below is satisfied by all samples in the “Luminal A” cluster and by none of the non-Luminal A samples:

$$\begin{array}{l} P_{A}=[\text{Expression of}\;GATA3\geq 0.49].\text{AND}.\\ \left[\text{Expression of }Liv-1\geq -0.25\right] \end{array}$$

**Figure 3:**
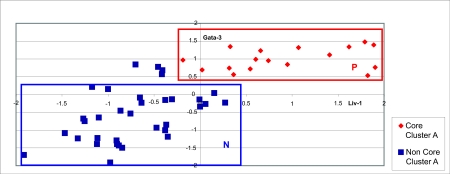
An example of a pattern (pattern ***PA***) characteristic of the Luminal A core cluster (Cluster A) and an example of a pattern (pattern ***NA*** ) characteristic of the non-Luminal A cases. Notice that ***P*** is satisfi ed by all the samples in the Luminal A group, while ***N*** is satisfi ed by 88% of the non-Luminal A cases. Both patterns ***P*** and ***N*** are expressed as bounding constraints on the expressions of genes Liv-1 and Gata-3.

For illustration, Figure 3 shows two patterns ***PA*** and ***NA***, in the 2-d expression plane for *GATA*3 and *Liv*-1.

A pattern is characterized by its degree, prevalence, and homogeneity. The *degree* is the number of genes appearing in its defining conditions. The *prevalence* of a pattern is the percent of positive (negative) cases which satisfy the pattern. The *homogeneity* of a pattern is the percentage of positive (negative) cases covered by it. In general, patterns useful for classification have low degree and high prevalence and homogeneity.

We identified all patterns for the 60 core samples over the selected 148 genes by applying the combinatorial algorithm described in (Alexe and Hammer, 2005). Briefly, each sample from a core cluster was placed in a box by defining cuts in gene expression space which distinguish it from the samples belonging to other core clusters. The boxes were then merged by extending them along all possible dimensions without allowing any member of the opposite class to be included in the box. The maximal boxes so obtained defined the patterns.

The pattern parameters (degree, prevalence, and homogeneity) were determined by estimating the classification accuracy of a weighted-voting model constructed on pattern data through 10-fold cross-validation experiments. Pattern-based weighted voting is a meta-classification scheme in which individual patterns are “voters” for a phenotype. The performance of a multi-pattern meta-classification system is better than the performance of single patterns if the patterns are uncorrelated (Merz, 1998). Uncorrelated patterns were selected by requiring the patterns to be defined on non-overlapping subsets of features. To avoid over-fitting, the patterns were required to use no more than five genes each.

We found many patterns of degree 2 and 3 for each phenotype, each of which was common to more than 90% of the samples in the cores. Table 3 presents some of these patterns. The striking feature of Table 3 is that simple conditions on a few genes are able to generate a very clean classification in the cores. *Several genes occurred frequently in the patterns, suggesting an active association with disease*. For example, KIAA1691, PREP, CX3CL1, LIV-1, PLOD, GATA-3 occur in 20% of patterns for Luminal A, while PRAME, PLAT, CCNE1, FKHL7, clone MGC:22588 IMAGE:4696566, occur in 15% of the patterns for the Basal group. There are also several genes which are good uni-gene markers but are not found in patterns.

### Consistency of Core Assignments Using Either Patterns or Clustering

A positive pattern is a set of conditions satisfied by a sample that belongs to a core cluster. A negative pattern is a set of conditions satisfied by a sample that belongs to the complement of the core cluster. For each unlabeled sample we counted the number of positive minus the number of negative patterns satisfied by it for each core cluster. The sample was assigned to the core cluster for which the ratio obtained by dividing this number to the total number of patterns for the core cluster, was positive and maximum. If the maximum ratio was negative or if it was assigned to multiple core clusters then the sample remained unclassified (Alexe et al. 2005c). The classification of samples to cores was validated using leave-one-out experiments on patterns. Over the sixty samples in the cores, in each such experiment, the entire procedure (gene selection, pattern extraction and sample classification) was repeated sixty times, once for each omitted sample.

A comparison of our clustering and pattern assignments with the original classification is presented in Table 4. The color scheme is that if the sample is robustly assigned to a phenotype, its entry is the color of that phenotype. Samples whose classification is either poor or ambiguous are in black or left blank respectively. When the pattern and cluster classifiers agree, the assignment can be considered accurate. When they differ, no classification is possible. From a treatment perspective, the recommendation of such an inconclusive assignment would be retesting. The clustering and patterns classifiers for the unassigned samples in the Sorlie et al. paper are shown in Table 5. Some of these originally unassigned samples are assigned to a consistent phenotype by our methods.

Table 6 summarizes the sensitivity and specificity of the pattern based classifier showing once again the robustness of the classification into phenotypes Normal, Luminal A and Basal and the unreliability of the other two phenotype classifications.

### Validation on an External Dataset Data 2

We used the markers identified in Data 1 to classify samples in Data 2. These two datasets had 93 genes in common. Of these, 79 were in our 391 uni-gene set and a subset of 38 of these were in the smaller subset of 148 genes. Of the latter, 23 were markers for Luminal A, 4 were markers for Luminal B, 3 were markers for ERBB2+ and 12 were markers for the Basal group. For each of the 38 genes, we normalized the data sets relative to each other by equating the average intensity of each gene for the normal samples in the two data sets. In each dataset, the expression level of each gene was replaced with its quartile value across all samples. We recomputed a pattern-based classifier trained on the known core clusters in the Sorlie et al. (2003) data and used it to predict the phenotype for Ma et al. 2003 samples.

**Figure 4:**
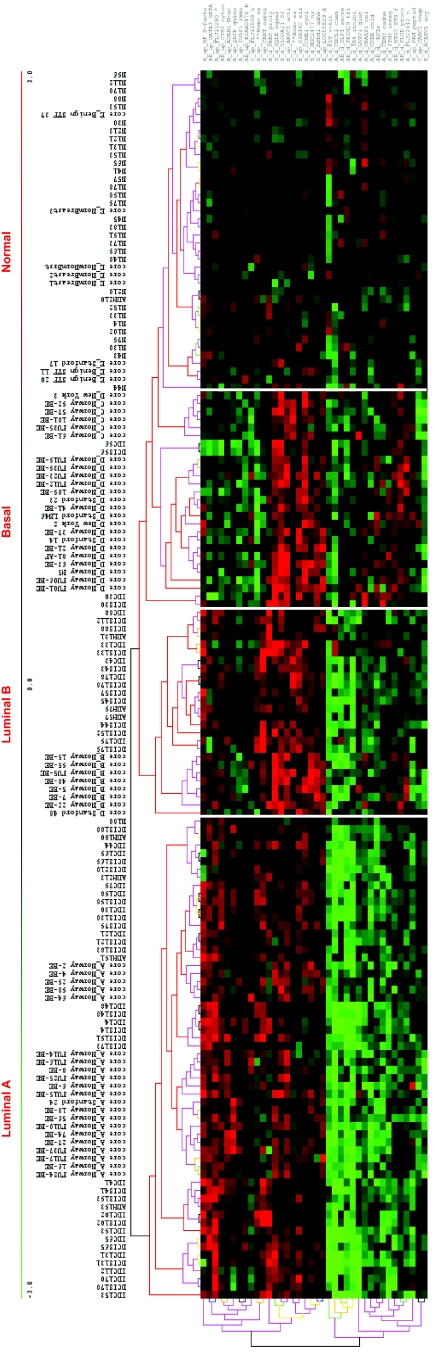
Heatmap of combined Ma et al. and Sorlie et al. data using the 38 genes identified in the latter data. There are four distinct clusters which are separtaed by vertical lines in the plot. The Normals, Luminal A and Basal core samples from Sorlie et al. cluster well enough with samples in the Ma et al. data to make a phenotype identifi cation possible for the latter data. The B core cluster (Luminal B) looks similar to the Luminal A core cluster with some genes over expressed. Core cluster C (ERBB2+) is most similar to Core D (Basal) presumably because the discriminator gene ERBB2 gene is not on the Ma et al. chip set . The sample labels in the Ma et al. data indicate stages of disease (ADH, DCIS or IDC) and the index number of the patient. Notice that samples from the same patient, even if in different stages of BCA, cluster together.

Figure 4 shows a heat map of the 38 genes in common between the datasets. This plot includes all core samples from Data 1 and all samples from Data 2. The Normal samples from both sets cluster nicely showing that the global normalization was done correctly. The Luminal A cluster is easily identified because all Luminal A core samples from Data 1 cluster together with several samples from Data 2. There is also a distinct Basal cluster with most Data 1 Basal samples and a few Data 2 samples on its edges. Finally, there is another cluster with some Core B samples which looks quite similar to Luminal A. The core C samples are mixed in with the Basal cluster (as was already noticed in Figure 1c). We conclude that it is not possible to assign Luminal B or ERBB2+ phenotypes to samples in Data 2 based on Data 1 because a) There are very few genes in these categories (3/38 for ERBB2+ and 4/38 for Luminal B), b) the ERBB2 gene is missing in Data 2 and c) The quality of the patterns using the 38 genes for these two phenotypes is poor. Indeed, for core C, there are no patterns at all and for core B, the patterns are of poor statistical quality.

To further validate the consistency of our assignments, we trained a pattern-based classification model on quartile discretized Data 1 samples and used it to predict the phenotype for the samples in Data 2 using majority voting. When the prediction from patterns agreed with the prediction from clustering as in Figure 4, we felt confident of the diagnosis, otherwise not. Our predicted phenotypes for the Ma et al. data are given in Table 7.

### Pathways for each Core

To identify processes/pathways that are common and particular to the different phenotypes, we used the bioinformatics public resources DAVID (Dennis et al. 2003), BioRag (Pandey et al. 2004), iHOP (Hoffmann and Valencia, 2004) and BRB Tools (http://linus.nci.nih.gov/BRB-ArrayTools.html). The method used for GO functional class scoring is given in Supplementary Information II.

Table 8 is a detailed explanation of some of the 148 uni-gene biomarkers identified for each core (see also Tables 2a – d). Table 9 presents the GO categories enriched for the genes associated with the cores. The statistical significance of the enriched GO categories is computed as described in Supplementary Information II. The complete list of gene markers for the core phenotypes involved in the enriched GO categories is available in Supplementary Table 4.

Whereas we discuss markers for each core subtype, we have strong confidence only in the markers for Luminal A and Basal.

In Luminal A, ESR1 is up-regulated, indicating that the estrogen receptor pathway is turned on.

The KIT gene was already known to be lost in breast cancer. Introduction of the c-kit gene leads to growth suppression of a breast cancer cell line, MCF-7 (Nishida et al. 1996). The Neuregulin 1 gene, which is up-regulated, is a direct ligand for ERBB3 and ERBB4, and an indirect activator of ERBB2, though the ERBB2+ subtype is identified with Cluster C. The nuclease sensitive element binding protein (NSEP1), which is also up-regulated, is known to inhibit p53 induced apoptosis (Zhang et al. 2003). It has also been recently shown to be a target of Akt phosphorylation, and that disruption of phosphorylation inhibits tumor growth (Sutherland et al. 2005). This gene is involved in D4-GDI signaling pathway, which may also be up-regulated.

A number of Luminal A markers were previously identified cancer related genes. The ID4 gene, which was also reported to be down-regulated in gastric adenocarcinoma and leukemia, may cause the alteration of the TGF-beta signaling pathway which regulates the growth and proliferation of cells, blocking the growth of many different cell types. The TGF-beta receptor includes Type I and Type II subunits that are serine-threonine kinases that signal through the Smad family of proteins. Another cancer related gene is GSTP1, which was reported to be lost in different types of cancers including prostate cancer, lung cancer and squamous cell carcinoma. Other cancer related genes include the TFF3 gene, which was shown to activate STAT3, (an oncogene) signaling in human colonic cancers (Rivat et al. 2005) and the VEGF receptor FLT1 gene.

Other Luminal A marker genes include up-regulated immune system related genes (SLPI , BF, and C4B), anti-apoptotic gene ASAH1; collagen related gene PLOD and actin gamma 2 gene. Other genes constitute mostly metabolic genes (with a significant enrichment, see Table 9), including fructose-1,6-bisphosphatase 1 (FBP1), glutamate dehydrogenase 1 (GLUD1) and acyl-Coenzyme A dehydrogenase (ACADSB).

Biomarkers for Cluster B (Luminal B) include fibroblast growth factor FGFR4 which might be from the fact that this family of genes is known to be overexpressed in cancers of the cervix and bladder, though their role in breast cancers is more controversial (Streit et al. 2004; Jezequel et al. 2004); two cancer related genes: Gammaglutamyl hydrolase (GGH) gene, which was also identified as a biomarker for pulmonary neuroendocrine tumors (He et al. 2004), and laminin, gamma 2 (LAMC2) gene, which was reported to be involved in tumor invasion and metastases in pancreatic ductal adenocarcinoma (Takahashi et al. 2002) and endometrial adenocarcinomas (Maatta et al. 2004). The latter gene is down-regulated in the breast cancer data sets analyzed here.

Generally, Cluster C (ERBB2+ subtype) biomarkers appear to be mostly receptors, receptor binding proteins and signal transduction related proteins (Table 9). As expected, the most characteristic of these genes is the up-regulated ERBB2 gene. Other important genes include two breast cancer related genes, namely, the F2R gene, a matrix metalloprotease-1 receptor that promotes invasion and tumorigenesis of breast cancer cells (Boire et al. 2005); and PPAR binding protein, coactivator of ESR1 and overexpressed in breast cancer (Zhu et al. 1999). The down-regulation of FLNB filamin B alters the MAP Kinase pathway with implications in both growth control and development.

The marker genes for the Basal phenotype (Cluster D) are significantly involved in cell cycle, regulation of cell proliferation, endoplasmic reticulum as well as in various metabolic processes. Important cancer related genes identified for this phenotype are CDK6 gene, which inhibits proliferation of human mammary epithelial cells (Lucas et al. 2004); SIAT4C, which is down-regulated in RCC (Saito et al. 2002), RHOB, which is known to be a pro-apoptotic and tumor suppressor gene, and the FLT1 and TFF3 gene. Plasminogen activator gene (PLAT) is involved in tissue remodeling while fibromodulin (FMOD) gene has a primary role in collagen fibrillogenesis.

The last of the clusters is the control or normal group. Here we find that the genes identified as significant markers are involved in organelle organization and biogenesis, cytoskeleton organization and biogenesis, or in metabolic pathways (e.g. cofactor biosynthesis). These represent genes that are pathologically expressed in all tumor strata; consequently they are able to robustly stratify BCA samples from control (Normals).

Overall, the biomarkers notably constitute genes that participate in breast cancer related pathways (e.g. marker genes involved in estrogen receptor pathway) and genes that were previously implicated in other cancer types (e.g. GSTP1, FLT1, see Table 8). Moreover, the enriched categories in each phenotype are biologically plausible, having already been implicated in cancer transformation (e.g. cell cycle, cell motility, cytoskeleton organization) (Hanahan and Weinberg, 2000) or being potentially important in transformation (signal transduction pathways, metabolism).

## Summary and Discussion

We have presented a robust clustering and pattern based analysis of the phenotypes identified by Sorlie et al. 2003. We find that the clusters for Luminal A, Basal and Normal subtypes are homogenous and have predictive content. However, the Luminal B and ERBB2+ assignments are sensitive to data perturbations. One reason for this is that the genes chosen for the classification are too few and not appropriate for these two categories. This is evidenced by the fact that the number of genes for Luminal B and ERBB2+ that pass our stringent robustness filters is small. Another reason is that hierarchical clustering is inappropriate to resolve the subtleties of the Luminal B and ERBB2+ categories. Finally, these subtypes are more heterogeneous than Luminal A and Basal and possibly have further substructure not classifiable with the genes in this dataset. A larger number of samples and better/more genes are necessary to test these conclusions.

Several samples previously unclassified in Sorlie et al. 2003 were classifiable by our techniques. We also found several samples which show a complex (multiple) phenotype signature. Given the treatment implications, the patients from whom these samples were taken should undergo further analysis or different treatment.

We also describe a general method to deal with *sensitivity to noise* in gene array data, which often confounds the analysis. There are four principal sources of noise. The first, which we cannot do anything about, is the experiment itself: a) different samples handled differently in and experiment or between different labs; b) data improperly collected or improperly recorded/measured; c) microarray or cDNA readout with missing or unreliable entries. The second type of “noise” is stochastic noise; from statistical errors in the measurement of the signal or from normal variation within a phenotype in the sample population. We show how to partially account for this noise by data perturbations and consensus analysis. A third source of noise is the data analysis methods used. In particular, there are many different definitions of distance between gene expression vectors and many different clustering techniques. These often lead to different clusters depending on parameter choices, and to clusters that are unstable to perturbations. Our method robustly deals with this issue to get reliable predictions. A fourth source of noise derives from the genes selected as the basis for the analysis (Ein-Dor et al. 2005). This set results both from the initial choice of genes on the chip and the subset of genes that is used in the clustering. The choice of genes on chips will improve only if chip manufactures come up with better chips, possibly motivated by the biology of the underlying processes. However, given a gene set, this paper describes a procedure to select a data perturbation independent and predictive subset of the genes.

The fundamental requirement of any clustering analysis is the assignment of confidence levels to clusters. This is particularly important in gene expression analysis where a small sample set is clustered using a large set of noisy genes which makes the clustering results sensitive to noise and susceptible to over-fitting. Our methods use re-sampling and cross validation to simulate perturbations of the data, and this allows us assess the stability of the clustering with respect to sample variability.

In functional genomics, agglomerative hierarchical clustering (HC) has been widely adopted as the unsupervised analysis tool of choice, mainly because of its intuitive appeal and its visualization properties. By not committing to a specific number of clusters, HC provides for a multi-resolution view of the data that can be extremely useful in exploratory data analysis. However, the method does not provide for an “objective” criterion to establish the number of clusters and the clusters’ boundaries. Furthermore, the resulting trees are known to be highly unstable to small perturbations of the data. The trees also tend to preserve sample joining errors made at earlier stages.

To correct for these problems, we recommend averaging over perturbations of the original data. The hierarchical clustering algorithm can then be applied to each of the perturbed data sets, and the agreement, or consensus, among the multiple runs can be assessed. This technique will measure the “stability” of the discovered clusters to sampling variability. The basic assumption of the method is intuitively simple: if the data represent a sample of items drawn from distinct sub-populations, and if we were to observe a different sample drawn from the same subpopulations, the induced cluster composition and number should not be radically different. Therefore, the more the attained clusters are robust to sampling variability, the more confident we can be that these clusters represent real structure. Overall, the procedures suggested here will be of use in examining any data in a way that makes the predictions insensitive to stochastic and systematic variation.

A frequent concern in gene-array data and analysis is whether the data is reproducible, and whether the inferences are consistent with current biological knowledge. In this paper we address the first issue by applying the results of our analysis on one data set to make predictions on another. For the phenotypes which cluster well, we can make definite predictions on the unseen data. In addition, we identify pathways via genes whose markers are predictive of phenotype. It is likely that these genes have only diagnostic value, i.e. they are downstream effects of an established disease process whose cause is outside the identified set of genes. This is a problem with most microarray data which is usually available only for cells which show established disease.

## Supplementary Information

### Supplementary Information I: Multiple Testing Correction Metrics

**Table:** The general multiple hypothesis testing analysis used in our paper results in the following matrix

	# non–rejected hypotheses	#rejected hypotheses	
# true null	*U*	*V*	
hypotheses (non-diff. genes)		***Type I error***	***M*_0_**
# false null	*T*		
hypotheses (diff. genes)	***Type II error***	*S*	***M*_1_**

We use the following statistics to analyze this table.

*False discovery rate* (*FDR*). The FDR (Benjamini and Hochberg 1995) is the expected proportion of Type I errors among the rejected hypotheses: *FDR* = *E*(*Q*); with *Q* = *V/R* if *R* > 0 and *Q* = 0; if *R* = 0.

The *q-value* of a gene (Storey and Tibshirani, 2003) is defined as the minimal FDR at which it appears significant.

*Family–wise error rate* (FWER, Dudoit et al. 2003). The FWER is defined as the probability of at least one Type I error (false positive): *FWER* = Pr(*V* > 0)

*The Bonferroni correction* (Bonferroni 1935) : Suppose we conduct a hypothesis test for each gene *g* = 1,…,*N*, producing an observed test statistic: *T*_g_ , an unadjusted *p*–value: *p_g_*. = the probability under the null hypothesis that the test statistic is at least as extreme as *T_g_*. Under the null hypothesis, Pr(*p_g_* < *a* ) = *a*.

Bonferroni adjusted p–values: **p**_g_ = min (1, N p_g_.)

References for Supplementary Information IIIBenjaminiYHochbergY1995Controlling the false discovery rate: a practical and powerful approach to multiple testingJ. Roy. Statist. Soc. Ser. B57289300BonferroniCE1935Il calcolo delle assicurazioni su gruppi di testeIn Studi in Onore del Professore Salvatore Ortu CarboniRome: Italy1360DudoitSPopper ShafferJBoldrickJC2003Multiple hypothesis testing in microarray experimentsStatistical Science1871103StoreyJDTibshiraniR2003Statistical significance for genome-wide studiesProc. Natl. Acad. Sci. U.S.A100944051288300510.1073/pnas.1530509100PMC170937

### Supplementary Information II: Functional class scoring for GO categories

We computed the statistical significance of a GO category within a collection of *N* gene markers by following Pavlidis et al. 2004: A *p*-value was computed for each of the *N* marker genes in our collection. Next, the set of *p*-values was tested for enrichment in a GO category by using the Functional Class (*LS*) and the Kolmogorov-Smirnov (*KS*) statistics. For a set of *N* genes, these are defined as

$$\begin{array}{l} LS=\sum_{i=1}^{N}(-\text{log}\;p_{i})/N\\ KS=\underset{i=1,\ldots ,N}{\text{max}}\frac{i}{N}-p_{i} \end{array}$$

The statistical significance of a GO category with *N* genes was measured by computing the empirical distribution of *LS* and *KS* from 100,000 random selections of *N* genes in the complete pool of genes. The *LS*/*KS* permutation *p*-value was computed by comparing the *LS*/*KS* statistics in these experiments to the measured value of these statistics for the selected genes. A GO category was considered enriched if its corresponding LS or KS re-sampling *p*-value was below 0.005.

References for Supplementary Information IIPavilidisPQinJArangoV2004Using the gene ontology for microarray data mining: A comparison of methods and application to age effects in human prefrontal cortexNeurochem. Res291213221517647810.1023/b:nere.0000023608.29741.45

## Figures and Tables

**Figure 1a:**
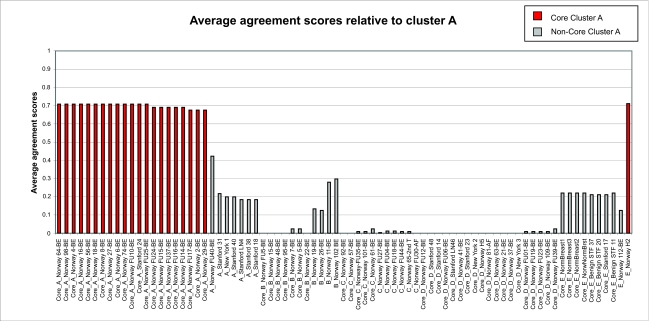
Average agreement scores relative to cluster A.

**Figure 1b:**
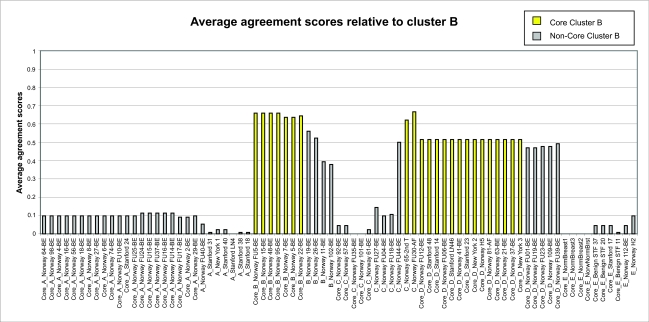
Average cluster agreement scores relative to cluster B.

**Figure 1c:**
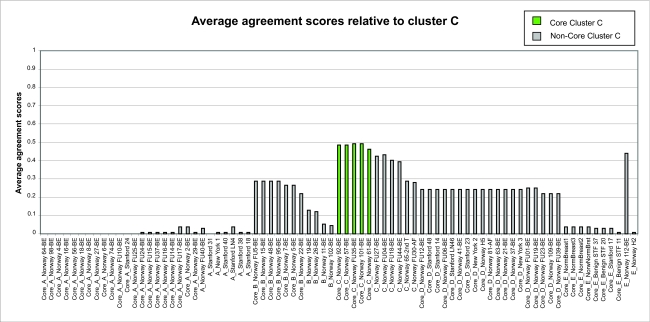
Average cluster agreement scores relative to cluster C.

**Figure 1d:**
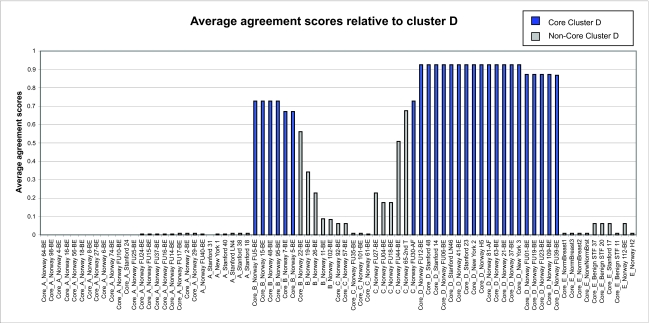
Average cluster agreement scores relative to cluster D.

**Figure 1e:**
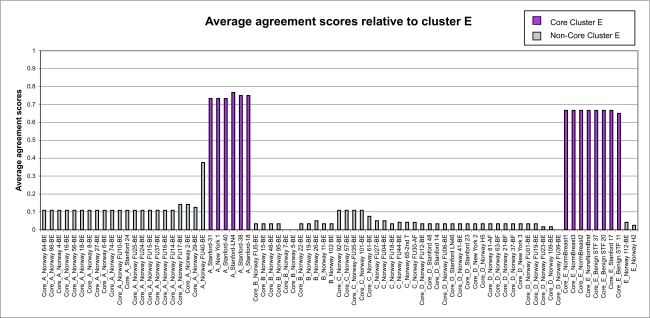
Average cluster agreement scores relative to cluster E.

**Figure 1f:**
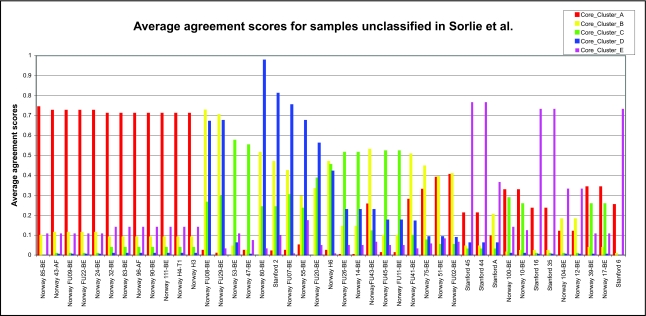
Agreement scores for the unclassifi ed samples in Sorlie et al.

**Table 1:**
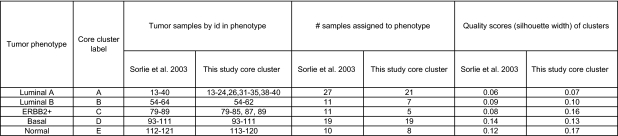
Summary of the classifi cations of tumor samples in the core samples (present study) and previous work. Sample identifi cation numbers refer to the original data of Sorlie et al. 2003. The numbers of samples assigned to each phenotype by the original classifi cation and by our clustering are shown in columns 5 and 6. We see that a larger fraction of assignments into the phenotypes Normal, Luminal A and Basal are correct. The silhouette scores are given in columns 7 and 8.

**Table 2a:**
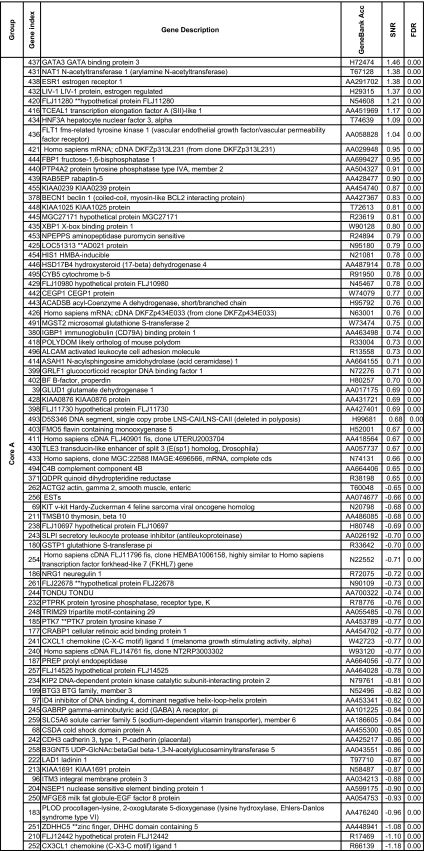
Collection of uni-gene markers for the Luminal A phenotype. The markers are sorted in decreasing order with respect to to the signal-to-noise ratio.

**Table 2b:**
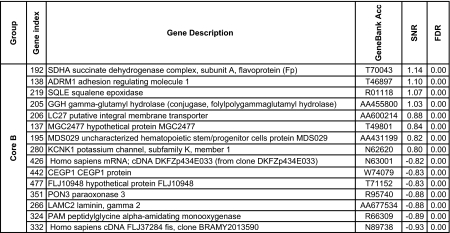
Collection of uni-gene markers for the Luminal B phenotype. The markers are sorted in decreasing order with respect to to the signal-to-noise ratio.

**Table 2c:**
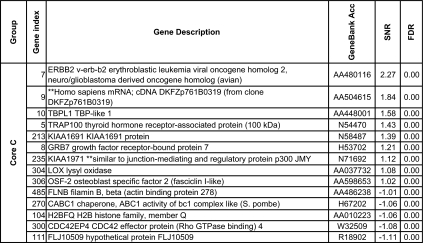
Collection of uni-gene markers for the ERBB2+ phenotype. The markers are sorted in decreasing order with respect to to the signal-to-noise ratio.

**Table 2d:**
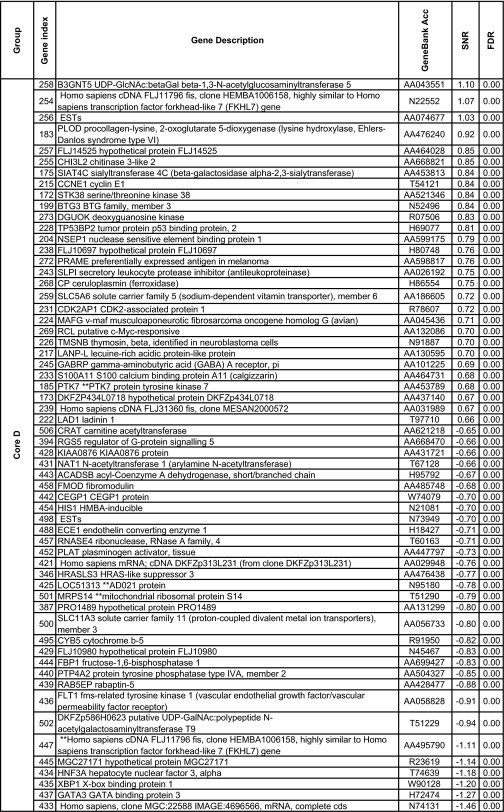
Collection of uni-gene markers for the Basal phenotype. The markers are sorted in decreasing order with respect to to the signal-to-noise ratio.

**Table 2e:**
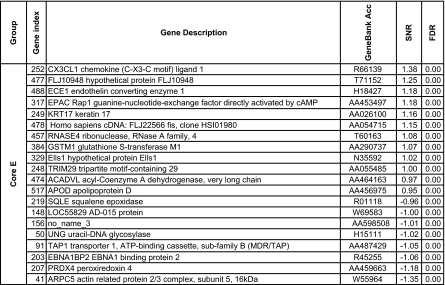
Collection of uni-gene markers for the Normal phenotype. The markers are sorted in decreasing order with respect to to the signal-to-noise ratio.

**Table 3:**
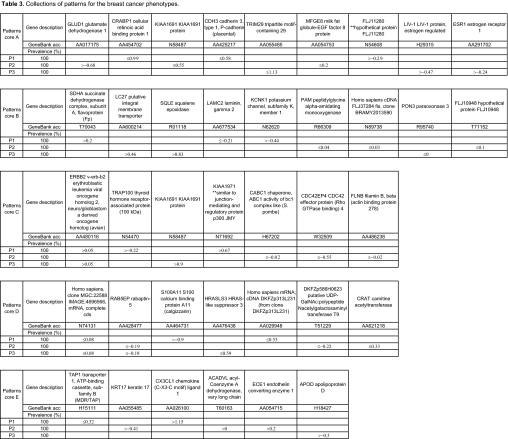
Collections of patterns for the breast cancer phenotypes.

**Table 4:**
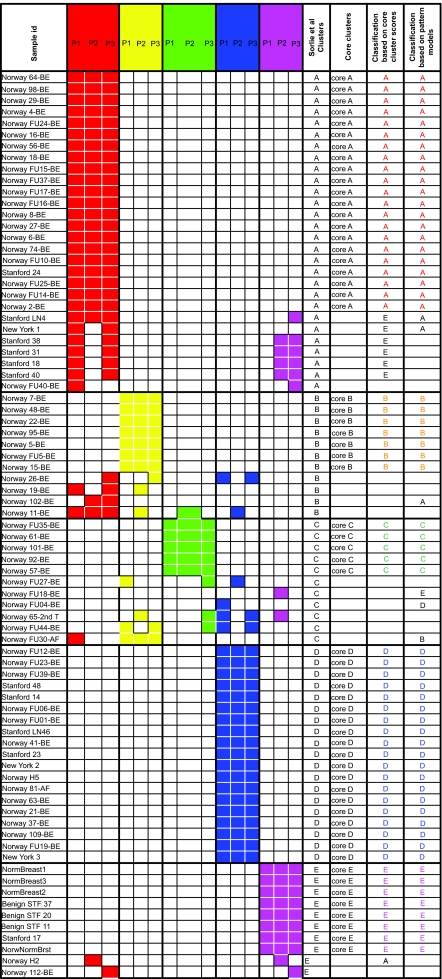
Phenotype classifi cation of breast cancer based on core clusters and pattern scores.

**Table 5:**
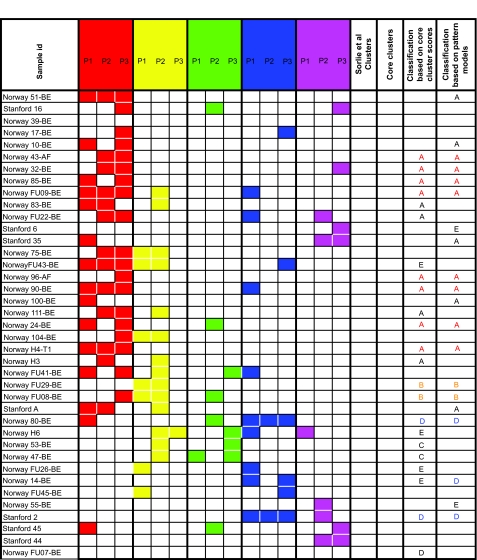
Phenotype prediction for previously unassigned breast cancer samples.

**Table 6:**
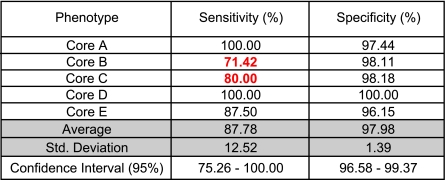
Classifi cation accuracy of pattern models through leave-one-out cross validation experiments.

**Table 7:**
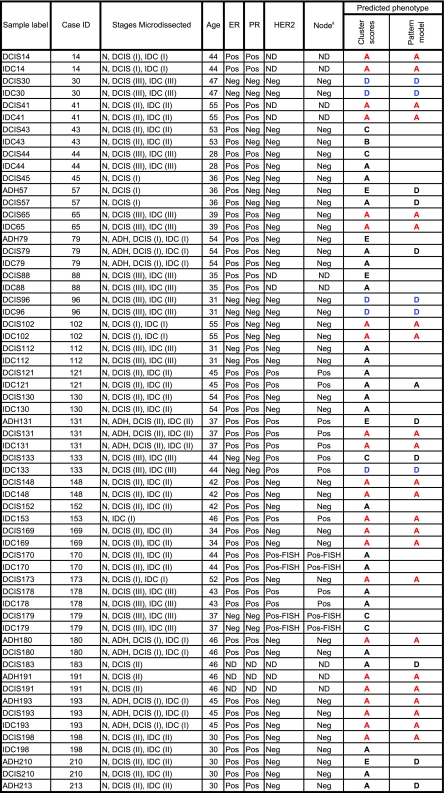
Predicted phenotype for samples in Ma et al. data using patterns from core clusters in Sorlie et al. 2003. We are confident of the phenotype assignment for those samples marked in color in columns 9 and 10.

**Table 8:**
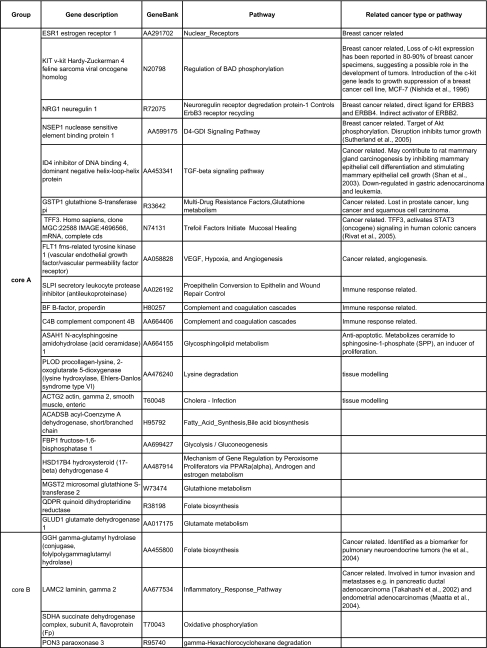

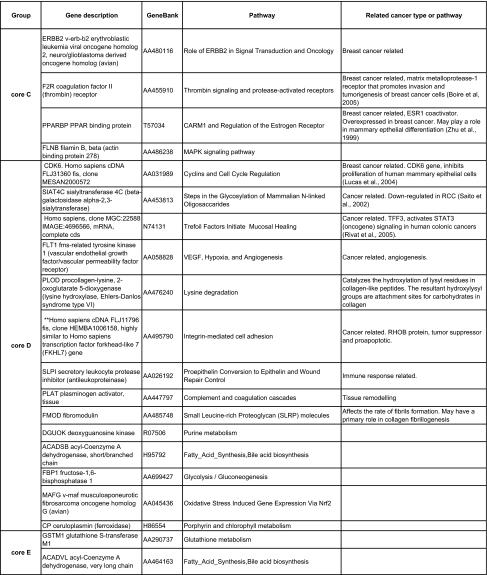
A complete listing of the associated pathways for the biomarkers available in different databases on the web (BIOCARTA, KEGG, GENMAPP).

**Table 9:**
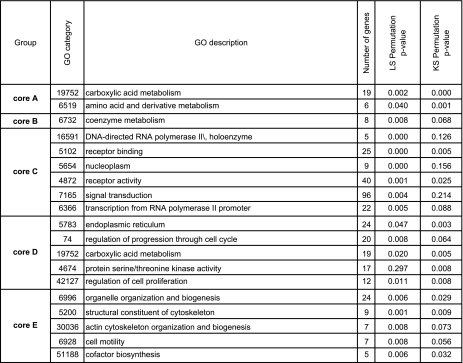
Enriched GO properties for the core phenotypes.
